# The combined prevalence of classified rare rheumatic diseases is almost double that of ankylosing spondylitis

**DOI:** 10.1186/s13023-021-01945-8

**Published:** 2021-07-22

**Authors:** Judith Leyens, Tim Th. A. Bender, Martin Mücke, Christiane Stieber, Dmitrij Kravchenko, Christian Dernbach, Matthias F. Seidel

**Affiliations:** 1 Center for Rare Diseases Bonn (ZSEB), University Hospital, Bonn, Germany; 2 Department of Neonatology and Pediatric Care, Children’s University Hospital, Bonn, Germany; 3 Institute of Human Genetics, University Hospital, Bonn, Germany; 4 Institute of General Practice and Family Medicine, University Hospital, Venusberg-Campus 1, 53127 Bonn, Germany; 5 Department of Radiology, University Hospital, Bonn, Germany; 6 Division of Medical Psychology and Department of Psychiatry, University Hospital, Bonn, Germany; 7 Department of Rheumatology, Spitalzentrum-Centre hospitalier, Biel-Bienne, Switzerland

**Keywords:** Rheumatology, Rare diseases, Epidemiology, Vasculitis, Arthritis, Myositis, Fever

## Abstract

**Background:**

Rare diseases (RDs) affect less than 5/10,000 people in Europe and fewer than 200,000 individuals in the United States. In rheumatology, RDs are heterogeneous and lack systemic classification. Clinical courses involve a variety of diverse symptoms, and patients may be misdiagnosed and not receive appropriate treatment. The objective of this study was to identify and classify some of the most important RDs in rheumatology. We also attempted to determine their combined prevalence to more precisely define this area of rheumatology and increase awareness of RDs in healthcare systems. We conducted a comprehensive literature search and analyzed each disease for the specified criteria, such as clinical symptoms, treatment regimens, prognoses, and point prevalences. If no epidemiological data were available, we estimated the prevalence as 1/1,000,000. The total point prevalence for all RDs in rheumatology was estimated as the sum of the individually determined prevalences.

**Results:**

A total of 76 syndromes and diseases were identified, including vasculitis/vasculopathy (n = 15), arthritis/arthropathy (n = 11), autoinflammatory syndromes (n = 11), myositis (n = 9), bone disorders (n = 11), connective tissue diseases (n = 8), overgrowth syndromes (n = 3), and others (n = 8). Out of the 76 diseases, 61 (80%) are classified as chronic, with a remitting-relapsing course in 27 cases (35%) upon adequate treatment. Another 34 (45%) diseases were predominantly progressive and difficult to control. Corticosteroids are a therapeutic option in 49 (64%) syndromes. Mortality is variable and could not be determined precisely. Epidemiological studies and prevalence data were available for 33 syndromes and diseases. For an additional eight diseases, only incidence data were accessible. The summed prevalence of all RDs was 28.8/10,000.

**Conclusions:**

RDs in rheumatology are frequently chronic, progressive, and present variable symptoms. Treatment options are often restricted to corticosteroids, presumably because of the scarcity of randomized controlled trials. The estimated combined prevalence is significant and almost double that of ankylosing spondylitis (18/10,000). Thus, healthcare systems should assign RDs similar importance as any other common disease in rheumatology.

## Background

Rare diseases (RDs) are a complex problem in medicine, and the definition of a RD varies around the world. The European Union (EU) defines a disease as rare when it affects less than 5 people in 10,000 living in the EU, which translates to approximately 370,500 individuals being affected. In the United States, the Rare Diseases Act of 2002 defined a disease as rare when it affects less than 200,000 people. Patients frequently remain undiagnosed for many years, and treatment is often inefficacious. Data on prevalence, disease burden, treatment regimens, access to healthcare systems, and mortality are, to a great extent, unknown. Thus, RDs remain an unresolved challenge in modern medicine.

The classification, overall prevalence, and treatment options of rare rheumatic diseases are poorly defined. Disorders may affect the musculoskeletal apparatus with arthritis and myalgia but also involve other tissues in the form of myositis, vasculitis, autoimmune organ involvement, or bone diseases. Patients are often diagnosed as having a psychosomatic disorder due to missing or unrecognized somatic and/or objective findings. Patients not only have to cope with their disease burden, but are also at risk of developing additional psychiatric comorbidities. For example, patients with undiagnosed diseases in Germany have a prevalence of depressive symptoms three times higher than the average population (22% vs. 8.1%) [1, 2]. On the other hand, a verified diagnosis may aid the patient in accepting their diagnosis and coping with the ensuing symptoms and challenges. However, due to the scarcity of randomized controlled trials, treatment options for RDs are often limited and remain empirical. In addition, the definitions of key symptoms often vary in clinical studies, hampering uniform analyses.

RDs in rheumatology may be analyzed systematically by prevalence, genetic background and pathogenesis, clinical involvement, treatment options, and prognosis. Prevalence data vary according to age, as well as global and ethnic background. For example, seronegative symmetrical synovitis with pitting edema (RS3PE) has a high prevalence among the elderly, with 0.09% of all individuals over the age of 50 years being affected in Japan [3], but seems to be a quite rare disorder among younger individuals. Similarly, the prevalence of adult onset Still’s disease (AOSD) varies globally: 3.4–6.9/100,000 in Norway [4], 6.77/100,000 in Turkey [5], and 3.9/100,000 in Japan [6]. With respect to ethnic background, the estimated worldwide prevalence of Gaucher’s disease is 1–2/100,000, but in Ashkenazi-Jews the prevalence may be as high as 1/850 [7].

The pathogenesis and genetic backgrounds of some RDs in rheumatology have been studied increasingly in recent years, and in some cases well elucidated. Blau syndrome was described in 2001 and is characterized by mutations in the *CARD15/NOD2* gene [8] and overexpression of autoinflammatory cytokines [9]. Interestingly, mutations in *CARD15/NOD2* are also associated with other diseases with inflammatory involvement, such as Crohn´s disease and arthritis [10]. Familial cold urticaria (FCU), Muckle-Wells syndrome (MWS), and neonatal-onset multisystem inflammatory disease (NOMID or chronic infantile neurologic cutaneous articular syndrome [CINCA]) were originally thought to be three similar but distinct diseases. Further evidence has shown that all three syndromes are the result of mutations in the same gene, *CIAS1*. They are now referred to as different phenotypes of the same disorder, namely cryoporin-associated periodic syndrome (CAPS) [11, 12]. The MEFV gene, best known for causing familial Mediterranean fever (FMF) equally demonstrates the importance of genetics in RDs [13, 14]. Heterozygous mutations in *MEFV* are also found in many children with periodic fever, stomatitis, pharyngitis, adenitis (PFAPA) [15, 16]. Findings suggest that exon variants in *MEFV* may also be associated with AOSD [17].

With respect to pathogenesis, infectious agents may also play a role in RDs. As some diseases follow a distinct seasonal pattern, infectious pathogenesis has been suggested for Kawasaki disease [18, 19], and IgA-vasculitis (formerly Henoch-Schönlein purpura) [20, 21].

Furthermore, some diseases are probably modulated by hormonal alterations, such as pachydermoperiostosis [22]. Although a disease-causing genetic mutation has been detected [23], males are seemingly more commonly and severely affected [24]. RDs in rheumatology involve a variety of tissues and organ systems. For example, joints are affected by pigmented villonodular synovitis [25], the skeletal system by Camurati-Engelmann disease [26], and internal organs by AOSD [27]. The skin is involved in pyogenic arthritis, pyoderma gangrenosum, acne (PAPA) syndrome [28], blood vessels in granulomatosis with polyangiitis (GPA) [29], connective tissue in systemic sclerosis [30], and muscles in inclusion body myositis (IBM) [31].

Treatment options often, but not exclusively, include corticosteroids, such as in RS3PE [32] and eosinophilic-myalgia syndrome [33]. Although research on RDs is often limited to retrospective, single center trials or case reports only, randomized controlled trials (RCTs) have been increasingly available in recent years for some conditions, such as granulomatosis with polyangiitis [34], PFAPA syndrome [35], and FMF [36].

The disease course may be classified as self-limited (e.g., Kawasaki disease), chronic with a variable remitting-relapsing course during treatment (e.g., Takayasu arteritis), and chronic with a predominantly progressive and difficult to control course (e.g., systemic sclerosis).

The prognosis for RD varies and may be affected by the primary disease, complications, and treatment, especially long-term immunosuppression.

The objective of this study was to analyze the complexity of RDs in rheumatology. Based on data from the literature, we identified some of the most important sets of rheumatic diseases and calculated their combined prevalence. Our data may give better insight into this area of rheumatology, aid specialized centers for RDs, and raise overall awareness in the healthcare field.

## Results

The diseases extracted from the databases are summarized in Table 2 of Appendix. The 76 syndromes and diseases were classified as follows: vasculitis/vasculopathy (n = 15), arthritis/arthropathy (n = 11), autoinflammatory syndromes (n = 11), myositis (n = 9), bone disorders (n = 11), connective tissue diseases (n = 8), which include inflammatory as well as non-inflammatory conditions, overgrowth syndromes (n = 3), and others (n = 8). A definitive genetic cause was identified in 26 diseases (34%). Out of the 76 conditions, 34 diseases (44%) were classified as chronic, primarily progressive, and difficult to control. Twenty-seven diseases (35%) were classified as chronic with a variable remitting-relapsing course. Six diseases (7%) were classified as self-limited. Acute phase reactants may be elevated in 49 diseases (64%). Corticosteroids are used as a therapeutic option in 49 diseases (64%). The mortality was variable and could not be determined precisely, but nine diseases (11%) were considered severe and potentially lethal if left untreated.

**Fig. 1:**
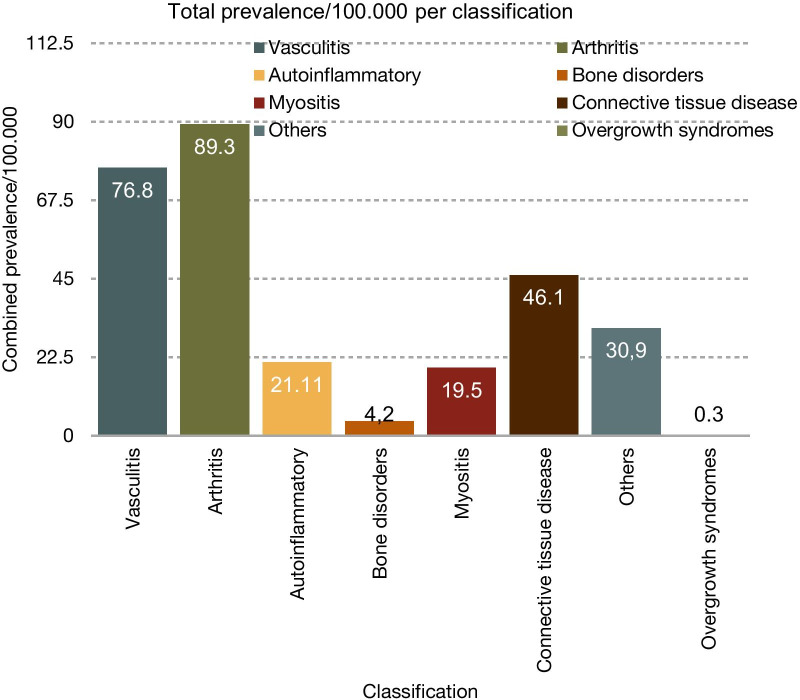
Overview of the total number of diseases in each classification

**Fig. 2:**
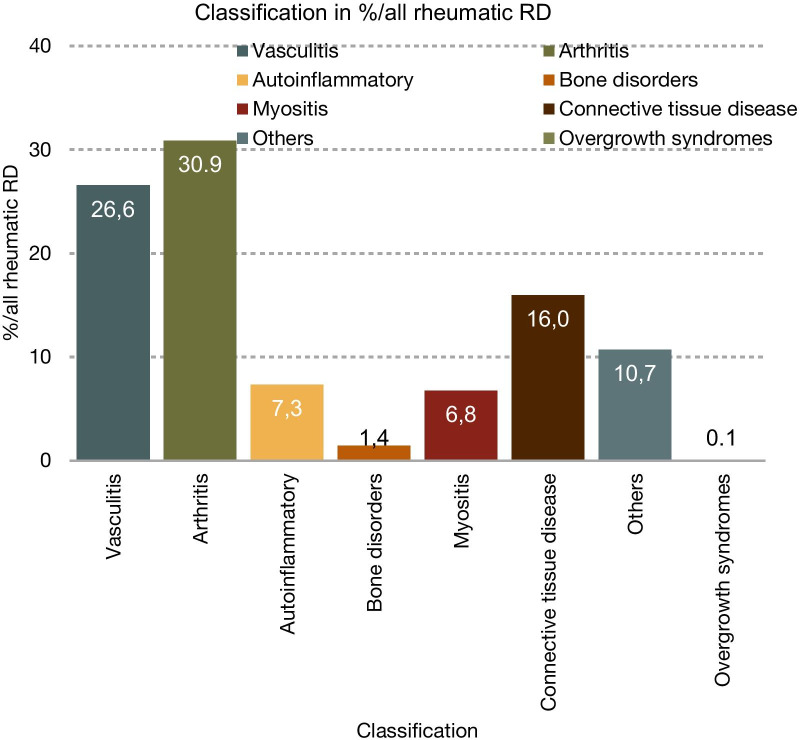
Flow chart of syndrome inclusion in the analysis. After conducting the literature search and analysis with regard to prevalence, four diseases were excluded due to high prevalence, and seven diseases were merged into three, resulting in a total of 76 syndromes included in further analysis

Prevalence data were available for 28 syndromes and diseases. For an additional five diseases, estimated prevalence data were already available. For another eight diseases, only incidence data were available. The prevalence of 38 diseases was estimated at 1/1,000,000, for 4 diseases at 1/100,000, and for 1 disease at 1/10,000. The combined prevalence per 10,000 is given in Table 1 (see also Figs. 1 and 2). The summed prevalence of all available and estimated RDs was 28.8/10,000.

## Discussion

RDs are challenging for patients, healthcare professionals, and societies. Signs and symptoms often remain unrecognized and patients are excluded from specific treatment. Patients are frequently diagnosed with a psychosomatic disorder. Conversely, some patients with true extrasomatic diagnoses insist on the presence of a RD and cause significant expenses to healthcare systems. To overcome this bias, the importance of RDs should be recognized in public healthcare.

The knowledge of RDs is mostly available from case reports or case studies. On one hand, these studies are important in order to document essential information, such as commonly reported symptoms, different treatment regimens, and outcomes. However, such studies may involve reporting bias and, thus, are difficult to compare. For example, multicentric histiocytosis is a disease primarily reported in Caucasian women. However, this may be due to increased awareness in Western countries. Furthermore, women may consult a doctor more often and simulate a female Caucasian predominance [37].

Most studies scrutinizing RDs are designed as single center, retrospective studies due to a lack of patient numbers or networks. Larger registries would provide an opportunity to conduct retrospective or prospective and multicenter studies with a greater number of participating patients. Therefore, further development of international expert centers and registries is in great demand. In recent years, advances have been made due to the establishment of international expert/reference centers. For example, the Eurofever project for autoinflammatory diseases provided classification criteria and evaluated treatment options for multiple disorders [38, 39].

In addition, genetic testing has become increasingly available and, thus, more important in recent years. For example, whole exome sequencing in patients with similar symptoms without previous knowledge of candidate genes led to the identification of *WISP 3* and *SLCO2A1* as the pathogenic mutations in progressive pseudorheumatoid dysplasia [40] and primary hypertrophic osteoarthropathy [23].

In this study, we aimed to identify and classify RDs in rheumatology. We were able to show that the most common symptoms in rare rheumatic diseases are arthritis (31.0%, 89.3/100,000), followed by vasculitis (26.6%, 76.8/100,000), and connective tissue involvement (16.0%, 46.1/100,000), which in this study includes inflammatory as well as non-inflammatory conditions. Importantly, the total prevalence of a symptom was commonly dominated by only a few comparatively prevalent diseases. For example, systemic sclerosis (prevalence: 22.5/100,000) makes up 48.8% of all rare rheumatic diseases with connective tissue involvement.

Our study has several limitations. First, the nomenclature for diseases and syndromes is often used interchangeably, and the same disease or a variation in the same group of diseases is sometimes named differently. For example, CAPS is formerly known as Muckle-Wells, CINCA/NOMID, or familial cold autoinflammatory syndrome [12]. This may lead to incomplete search results and impede the comparability of available studies. Second, most prevalence data are almost exclusively based on retrospective analyses of hospital information or questionnaires. Prevalence data also vary according to ethnic background, geography, and age, which makes the use of overall prevalence data quite uncertain. For example, FMF or Behçet´s are more common in Mediterranean and Middle Eastern populations [41, 42] and rare in other regions, demonstrating the difficulty in using a local geographic prevalence. Similarly, although currently considered a RD by the European definition, an Italian study found an unexpectedly high overall prevalence of 8.5/10,000 for cryoglobulinemic vasculitis, which would no longer be defined as a RD. Although this study had some limitations, including the implementation of a questionnaire leading to a higher participation rate in the elderly population than the younger age groups, we decided to exclude cryoglobulinemic vasculitis from our list of rare rheumatological diseases. Our reasoning for this was the methodically more accurate estimation of prevalence by this study [43]. A well-known, verified RD, systemic sclerosis is quite common in Choctaw Native Americans (prevalence 66/100,000 in Oklahoma Choctaws and 469/100,000 in full-blooded Choctaws [44]), but rare among all other studied populations [45]. This may be due to a unique HLA haplotype, but other environmental factors may also play a role. Furthermore, our estimates of prevalence data may be somewhat inaccurate. We probably underestimated the prevalence by choosing 1 in 1,000,000 rather than 1 in 100,000, and the total prevalence of rare rheumatic diseases is likely to be even higher than our estimate of 29.6/10,000.

Prevalence data may also differ depending on age. For example, giant cell arteritis is probably extremely rare in younger patients but quite frequent in patients older than 55 years of age (UK 250/100,000 [46]), with age-independent prevalence data being difficult to obtain. Because the overall estimated prevalence may be even higher than 1/10,000, we also excluded giant cell arteritis from our list of RDs in rheumatology.

Similarly, we also excluded systemic lupus erythematodes, one of the better known “rare” diseases in rheumatology from our study, as recent epidemiological studies suggest that it is probably not a rare disease by the above mentioned European definition [47]. Furthermore, we also excluded rhupus syndrome, because it is suspected to be an overlap of systemic lupus erythematodes and rheumatoid arthritis and thus its prevalence may be difficult to obtain and distinguish [48].

The classification system we used also has its limitations. Conditions can be classified by their etiology or by their clinical appearance. As the etiology of many rare diseases remains unknown, we decided to classify diseases according to their main organ system involved in the clinical appearance of the disease. Exceptions include the category of autoinflammatory and overgrowth syndromes, where multiple organ systems may be involved, and the conditions in one group have a basic (suspected) etiology in common. However, overlaps between included conditions may have occurred because of the complex nature of some RDs.

Another pitfall is that diagnostic and/or classification criteria may differ in varying definitions of the diseases (e.g., familial Mediterranean fever), are not well established, and have been suggested based on radiographic or histological findings. In most cases, no validation studies are available to confirm specificity and sensitivity.

We observed that RCTs are available only for some RDs, such as Behcet´s disease [49] or ANCA-associated vasculitis [34]. Furthermore, most follow-ups of patients with RDs are rarely published. Larger patient cohorts would be necessary to obtain reliable data on outcome, disease progression, morbidity, and mortality. Treatment complications in most, if not all, RDs may be due to the disease itself or adverse events to immunosuppressive treatment. These data merit in-depth analyses, as they may shed more light on the disease and its pathophysiology or potential treatment options.

Incidence and prevalence data have become available for increasingly more RDs and, in some cases, the prevalence has even increased in recent years (e.g., Kawasaki disease) [50]. This trend may be due to a true increase in incidence, increased diagnosis of previously undiagnosed patients due to an upsurge in physician or patient awareness (internet, patient support groups, RD associations, etc.), or simply due to better national reporting systems and databases. In addition, an increased overall life expectancy in the general population has led to an increased incidence and prevalence of conditions that predominantly affect the elderly (e.g., GCA). Continuously better treatment options may also lead to prolonged overall survival with an increase in prevalence rates.

In this study, we found that the cumulative prevalence of RDs in rheumatology is at least 28.8/10,000, which is almost double that of ankylosing spondylitis (18/10,000) [51], a rather common disease seen in practice. This observation suggests that symptoms should be carefully acknowledged in all patients, especially when an overt psychopathology is present, as many patients with a yet undiagnosed disorder show signs of depression or other psychosomatic disorders [1], which can obscure the differentiation between primary disease and secondary complication even more for the treating physician. Our study may aid physicians as a simple tool for diagnosing patients with an undiagnosed rheumatic disease by comparing the symptoms, prevalence, and likeliness of one disorder to another.

## Conclusion

Our study shows that RDs in rheumatology are not as rare as previously thought, affecting at least 28.8/10,000 people. Therefore, for every patient diagnosed with ankylosing spondylitis, 1.6 patients may suffer from a rare rheumatic disease. Although research and case reports of RDs are important, international expert centers are necessary to initiate and perpetuate patient cohorts and registries, establish classification/diagnostic criteria, and conduct clinical trials. Standard questionnaires and laboratory analyses may aid in obtaining better insight into the pathophysiology of RDs.

## Methods and materials

**Fig. 3:**
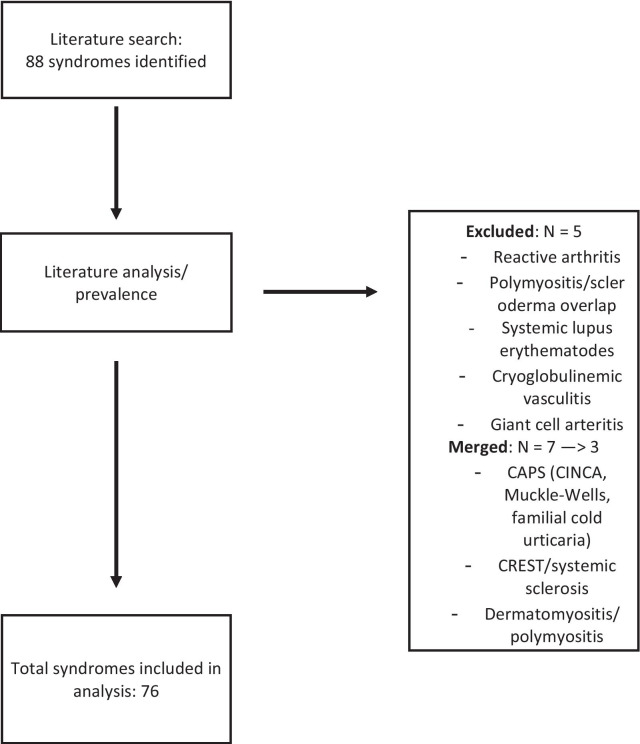
Overview of the combined prevalence per 100,000 per classification

The abstract archives of the European League Against Rheumatism, the American College of Rheumatology, Orpha.net, and the PubMed database were searched for the following terms: “rare” in combination with arthritis, arteritis, connective tissue disease, rheumatic, and vasculitis. Furthermore, terms were used in various combinations including arthralgia, autoimmune, fever, inflammation, joint pain, muscle pain, myalgia, and swollen joint. Identified syndromes were then classified according to their main appearance under the following terms: arthritis/arthropathy, bone disorders, autoinflammatory syndromes, connective tissue diseases, myositis/myopathy, overgrowth syndromes, vasculitis/vasculopathy, and others. Furthermore, we conducted a search of the literature and analyzed each disease according to the following criteria: prevalence, genetics/pathogenesis, diagnostic criteria, symptoms, laboratory findings, therapy, and prognosis. Diseases and syndromes were assessed for their overall prevalence and excluded if they did not meet the European definition of a RD (< 5/10,000) (see Fig. 3).

If more than one prevalence was available, the prevalence data were averaged accordingly.

For statistical reasons, we estimated the prevalence for diseases for which no epidemiological data were available as one of three possible values: < 1/1,000,000, 1/100,000 or 1/10,000. The overall total prevalence for all RDs in rheumatology was estimated by summing up the available or estimated individual prevalence of each disease.

## Data Availability

All data generated or analysed during this study are included in this published article. Data citations are included in the reference list.

## Appendix

See Table 1, 2.

**Table 1:** Results from the literature analyses

Classification	N =	Combined prevalence/100.000	%/All RD (prevalence)
Vasculitis	15	76.8	26.55
Arthritis	11	89.3	30.9
Autoinflammatory	11	21.105	7.32
Bone disorders	11	4.15	1.44
Myositis	9	19.5	6.76
Connective tissue disease	8	46.1	15.99
Others	8	30.89	10.72
Overgrowth syndromes	3	0.3	0.1
Total	76	288.15	100

**Table 2:** Overview of rare rheumatic diseases

Disease	Prevalence	Genetics/pathogenesis	Diagnostic criteria	Clinical features	Laboratory findings	Therapy	Prognosis/ Complications	Classification
Buerger's disease (thromboangiitis obliterans) [52–56]	Most common in Middle and Far East, estimated prevalence: 5/100,000 (Japan) 0.65/100,000 (Taiwan) → 2.8/100,000	Probably immune-mediated vasculitis, possibly associated with infectious agent; strong association with smoking; segmental occlusive inflammatory vasculitis (mainly small vessels, arteries and veins)	Many different diagnostic criteria (e.g., by Shionoya and Olin)	Disease onset in middle-aged, predominantly males; ischemic pain in extremities, numbness, skin ulcerations, thrombophlebitis, Raynaud´s phenomenon	Inflammatory markers usually normal	Smoking cessation, prostaglandin analogs, maximize blood supply, reduce risk of vasoconstriction (avoid coldness. etc.)	Remitting-relapsing, life expectancy usually normal, but morbidity increased (e.g., amputations)	Vasculitis/ vasculopathy
Eosinophilic granulomatosis with polyangiitis (EGPA, Churg-Strauss syndrome) [57–63]	17.8/1,000,000 (estimated, Japan) 10.7/1,000,000 (France) 14/1,000,000 (Sweden) → 1.4/100,000	Association with multiple different HLA genotypes, possibly IgG4-related disorder; Th-2 cells and eosinophils seem to play a major role in pathogenesis; small vessel necrotizing vasculitis	ACR criteria, Chapel Hill nomenclature definition	Disease onset in middle-aged males and females; asthma, weight loss, mononeuritis multiplex, non-erosive sinusitis/polyposis, skin lesions, lung infiltrates, pleural effusion, cardiomyopathy, glomerulonephritis	30 – 40% ANCA-positive (mainly MPO, but proteinase 3 also possible), eosinophilia; depending on organ involvement, elevated renal enzymes possible; elevated IgG4 can be found	Corticosteroids, immunosuppressants, rituximab	Chronic or remitting-relapsing, main complication with increased mortality is cardiomyopathy but overall mortality low	Vasculitis/ vasculopathy
Degos disease (malignant atrophic papulosis) [64–67]	Unknown, less than 200 cases reported, estimate < 1/1,000,000	Unknown; autosomal dominant inheritance discussed; thrombo-obliterative vasculopathy	Clinical and histopathological diagnosis	Onset usually at age 20–50 years, papular skin lesions with central atrophy and peripheral telangiectatic rim, sudden onset systemic involvement possible with high morbidity and mortality (bowel perforation, thrombosis, hemorrhage)	Coagulopathy in some patients	Anticoagulants, treprostinil, eculizumab	Limited and systemic variant, systemic variant has over 50% mortality within 2–3 years (due to bowel perforation and peritonitis)	Vasculitis/ vasculopathy
Hughes-Stovin syndrome (incomplete/ cardiovascular Behcet´s disease) [68, 69]	Unknown, ~ 40 cases described, estimate < 1/1,000,000	Unknown; angiodysplasia and infections discussed, maybe variant of Behcet´s disease; absence of oral/genital ulcers important for distinction	Clinical and radiographic diagnosis	Predominantly young males affected, multiple lung aneurysms cause cough, dyspnea, fever, chest pain, hemoptysis	Leukocytosis, anemia, elevated ESR and CRP	Corticosteroids, immunosuppressants, anticoagulant/ thrombolytic agents, surgery	Poor prognosis, massive hemoptysis and aneurysmal rupture are main causes of death	Vasculitis/ vasculopathy
Hypocomplementemic urticarial vasculitis (McDuffie syndrome) [70–74]	Unknown, estimate < 1/1,000,000	Mutations in *DNASE1L3* described, possible association with SLE; inflammation of dermal capillaries and postcapillary venules; possibly IgG4-related disease	Proposed criteria by Schwartz et al.	Predominantly in middle-aged females, chronic urticarial exanthema, angioedema, arthralgia/arthritis, ocular inflammation, glomerulonephritis, abdominal pain, angioedema, obstructive pulmonary disease	Low complement levels, anti-C1q-antibodies	Corticosteroids, immunosuppressants	Chronic, prognosis depending on systemic organ involvement (pulmonary, renal, cardiac), mortality low	Vasculitis/ vasculopathy
Kawasaki disease (mucocutaneous lymph node syndrome) [18, 75–77]	Prevalence unknown, incidence ranges from 3.7/100,000 (Australia) to 243/100,000 (Japan) for children < 5 years and increased in previous years, estimate < 1/100,000	Unknown, but genetic predisposition suspected (much more common in Asia), probably infectious trigger (seasonal peaks) causing small and medium vessel vasculitis	American Heart Association (AHA) guidelines, but primarily clinical diagnosis, as incomplete presentation is common	Predominantly young children until the age of 4 years affected with male predominance; conjunctivitis, exanthema, GI symptoms, fever, oropharynx involvement, lymphadenopathy, cracked lips, erythema of hand and feet, coronary aneurysm	Elevated CRP, ESR, leukocytosis, thrombocytosis	Intravenous immunoglobulins, high dose aspirin	Self-limited, good prognosis if treated, but coronary artery abnormalities occur in 25% if left untreated, leading cause of acquired heart disease in children in developed countries	Vasculitis/ vasculopathy
Leukocytoclastic/cutaneous small vessel/hypersensitivity vasculitis [78–80]	Prevalence unknown, incidence 4.5/1,000,000 estimate < 1/1,000,000	Unknown; more than 50% idiopathic, other possible causes include malignancy, autoimmune diseases, drugs (antibiotics, NSAIDs); neutrophilic inflammation in postcapillary venules	Chapel Hill criteria (histopathological)	Limited cutaneous variant with palpable purpura and lesions (often on lower extremity), or systemic organ involvement possible (most commonly renal)	Anemia, leukocytosis, elevated renal enzymes	Corticosteroids, immunosuppressants	Variable, commonly remitting-relapsing with treatment, overall survival good	Vasculitis/ vasculopathy
Microscopic polyangiitis (microscopic polyarteritis) [58, 59, 81, 82]	25.1/1,000,000 (France) 94/1,000,000 (Sweden) → 6/100,000	Unknown; possibly related to MHC II genes; environmental (silica) and autoimmune influence discussed; small vessel, necrotizing vasculitis with few or no immune deposits, primarily affecting kidneys and lungs	Chapel Hill criteria (histopathological)	Males slightly more commonly affected than females, onset primarily in elderly (age ≥ 60 years). Dyspnea, cough, hemoptysis, rapidly progressive glomerulonephritis, palpable purpura, GI symptoms, peripheral neuropathy	MPO-ANCA, microscopic hematuria	Corticosteroids, immunosuppressants (rituximab, cyclophosphamide)	Remitting-relapsing with treatment, poor prognosis without therapy, complications include end-stage renal disease, cardiovascular involvement, malignancy, and infections	Vasculitis/ vasculopathy
Behçet's disease [41, 83–85]	Estimated prevalence in Scandinavia: 2/100,000 Europe: 10.5/100,000 Mediterranean: 188/100,000 Others: 15.7/100,000 Not rare in Turkey (80–370/100,000), estimate 5/10,000	Association with HLA-B51; most common along the ancient silk road; infectious or environmental triggers; systemic occlusive vasculitis discussed	Many different criteria exist (e.g., New International Criteria of Behçet’s Disease)	Peak of disease onset in third decade of life (any age possible), recurrent eye inflammation (iridocyclitis/uveitis), oral and genital ulcers, skin manifestations (erythema nodosum, etc.), arthralgia, thrombosis, neurological symptoms, cardiac involvement	Pathergy-phenomenon, leukocytosis	Corticosteroids, colchicine, immunosuppressants, biologicals, small molecules, anticoagulants	Chronic disease with remitting-relapsing course, increased mortality in case of arterial and neurological involvement, possible cause of blindness	Vasculitis/ vasculopathy
Erythema induratum (Bazin disease, nodular vasculitis) [86, 87]	Unknown, estimate < 1/1,000,000	Unknown; type III or type IV hypersensitivity reaction suspected; associated with drugs (propylthiouracil), infectious (tuberculosis, hepatitis) or non-infectious diseases (leukemia, RA); diffuse panniculitis with neutrophilic vasculitis		Female predominance, recurrent nodules, usually on posterior lower legs, with focal ulceration and drainage, heal with scarring and post-inflammatory hyperpigmentation	Depending on underlying cause	Treatment of underlying cause, potassium iodide, NSAIDs, corticosteroids, immunosuppressants	Chronic, relapses are common	Vasculitis/ vasculopathy
Polyarteritis nodosa (Kussmaul-Maier disease) [58, 59, 88, 89]	30.7/1,000,000 (France) 31/1,000,000 (Sweden) → 3.1/100,000	Early-onset polyarteritis nodosa; mutations in *CECR1*, leading to deficiency in adenosine deaminase 2 (DADA2); other forms: idiopathic, cutaneous, and infection-associated (HBV); medium vessel, necrotizing vasculitis with segmental, mixed inflammatory infiltrates at branching points and microaneurysms (often in hepatic, renal, and mesenteric arteries), that spares the lung	Chapel Hill criteria (histopathological)	Male predominance, malaise, weight loss, fever, arthralgia, ulcers, livedo racemosa, myalgia, mononeuritis multiplex, purpura, GI symptoms, kidney infarctions, orchitis, hearing loss; no pulmonary involvement!	ESR and CRP elevated, leukocytosis, anemia, occasionally hypereosinophilia, hepatitis serology, liver enzymes, ANCA negative	Corticosteroids, immunosuppressant, biologicals (TNFi), antivirals, NSAIDs	Variable; chronic, acute, remitting-relapsing (10–20%) course possible; potentially life-threatening, remission can be achieved in many cases, good survival rate if treated early	Vasculitis/ vasculopathy
Primary central nervous system vasculitis (PACNS/primary angiitis of the CNS) [90–93]	Prevalence unknown, incidence: 2.4/1,000,000 estimate < 1/1,000,000	Different infectious agents suggested as triggers, segmental inflammation of CNS vessels	Proposed criteria by Calabrese and Mallek, histological criteria by Alrawi et al.	Disease most common in white males age ≥ 50 years, depending on localization different symptoms occur: headache, stroke, dementia, chronic meningitis, personality changes	Because of lack of systemic disease, inflammatory markers in blood are normal, but cerebrospinal fluid should be investigated	Corticosteroids + immunosuppressants (e.g., cyclophosphamide)	Chronic, controllable with medication, relapses common, fatal in the past, current mortality ~ 15%	Vasculitis/ vasculopathy
Henoch-Schonlein purpura (IgA vasculitis) [21, 94, 95]	Incidence 3–26.7/100,000 in children, 0.8–1.8/100,000 in adults, estimate 1/100,000	Unknown; several reports describe relationship to different HLA genes and MEFV gene mutations; infectious agent suggested in children (seasonal peak in fall and winter), in adults linked to cancer; small-vessel leukocytoclastic vasculitis	ACR criteria, criteria by Michet et al., EULAR pediatric criteria	Predominantly male children affected, purpuric rash, abdominal pain, joint pain, edema, renal involvement	Increased ESR, CRP, leukocytosis, anemia, proteinuria	NSAIDs, corticosteroids, immunoglobulins, immunosuppressants	Usually self-limited, but remitting-relapsing course possible, poor prognosis in case of renal involvement, worse prognosis in adults	Vasculitis/ vasculopathy
Takayasu arteritis [96–100]	2.82/100,000 (Korea) 4.7/1,000,000 (UK) 22/1,000,000 (Norway) 12.8/1,000,000 (Turkey) → 1.7/100,000	Different candidate genes: *HLA* variants, *FCGR2A/FCGR3A*, *IL12B*; more frequent in Asia; aortic and large vessel vasculitis	ACR criteria	Female predominance, fever, fatigue, weight loss, headache, differences in blood pressure of extremities, “pulseless disease”	Elevated CRP, ESR MMP-3 levels, PTX-3	Corticosteroids, immunosuppressants, biologicals (TNFi, IL-6i)	Chronic, good overall survival, cardiovascular disease is most common cause of death (infarction, thrombosis, etc.)	Vasculitis/ vasculopathy
Granulomatosis with polyangiitis (GPA, formerly Wegener granulomatosis) [29, 58, 59, 101]	160/1,000,000 (Sweden) 23.7/1,000,000 (France) → 9.2/100,000	Different genes suspected: *HLA* variants, *SERPINA1*; infectious, environmental (decreasing north–south gradient), and drug-induced triggers suspected; ANCA-associated, small vessel vasculitis	ACR criteria, Chapel Hill criteria (histopathological)	Malaise, myalgia, arthralgia, anorexia, weight loss, fever, oral ulcers, ear/nose/throat manifestations typical: eye manifestations (scleritis/uveitis, etc.), nasal discharge, epistaxis, upper airway obstructive disease	Elevated inflammatory markers, positive ANCA (proteinase 3 in 80%), urine analysis	Corticosteroids, immunosuppressants (rituximab, cyclophosphamide, azathioprine)	Chronic, relapses are very common, infections are main cause of death	Vasculitis/ vasculopathy
Adult-onset Still's disease (AOSD, Wissler's syndrome) [4–6, 102, 103]	3.4 – 6.9/100,000 (Norway) 6.77/100,000 (Turkey) 3.9/100,000 (Japan) → 5.3/100,000	Unknown; possibly related to *MIF*, HLA antigens, *MEFV*; different triggers suspected (infections, malignancies, medications, vaccinations)	Yamaguchi criteria, Fautrel criteria	Females slightly more affected, disease onset often at age 17–35 years, fever, maculopapular rash, arthralgia/arthritis (most commonly big joints), lymphadenopathy, hepatosplenomegaly, pleuritis, pericarditis, pneumonitis, eye involvement, abdominal pain, myalgia, alopecia, sore throat, weight loss, cranial nerve palsy	Leukocytosis, anemia, hypoalbuminemia, elevated liver enzymes, elevated ESR and CRP; ANA, ACPA and RF usually negative, elevated ferritin, mild proteinuria, increased IgE or elevated β2-microglobulin	NSAIDs, corticosteroids, DMARDs, immunosuppressants, biologicals (IL-1i, IL-6i)	Variable; 1/3 self-limiting, 1/3 relapsing, 1/3 chronic; overall survival good, complications include MAS	Arthritis/ arthropathy
Progressive pseudorheumatoid arthropathy of childhood (PPAC/ PPD/ PPRD/ SEDT-PA) [104–109]	Maybe not rare, but misdiagnosed as JIA; estimated prevalence < 1/1,000,000	Autosomal recessive; different mutations in *WISP3*; possible founder effect in Turkey; WISP mediates cell growth and differentiation in chondrocytes	Clinical and radiographic diagnosis with genetic confirmation	Disease onset usually in childhood (age 3–8 years), progressive joint stiffness, contractures, swelling of finger joints, osteopenia, slow linear growth, short stature, osteopenia, arthritis, difficulty in walking, genu valgum, hip pain, adult height usually below 3rd percentile, normal intelligence	No signs of systemic inflammation	Symptomatic: NSAIDs	Chronic, overall prognosis good because systemic organ involvement is absent	Arthritis/ arthropathy
Familial articular chondrocalcinosis (CPPD deposition disease/CCAL1 and CCAL2) [110–114]	Unknown, estimate < 1/1,000,000	Most cases autosomal dominant with variable penetrance; CCAL2: mutations in *ANKH*; CCAL1: chromosome 8 suspected, but gene not yet identified; increased extracellular pyrophosphate levels and formation of CPPD	Clinical and radiographic diagnosis	Disease onset in early childhood with calcium crystal joint deposition; arthritis/arthralgia, most commonly in knee and other big joints	Inflammatory markers may be elevated	Symptomatic: Corticosteroids, colchicine, NSAIDs	Chronic, usually not life-threatening, but high morbidity	Arthritis/ arthropathy
Pigmented villonodular synovitis [25, 115–118]	Incidence: 1.8/1,000,000 (USA), estimate < 1/1,000,000	Unknown; possible association with trauma/surgery, lipometabolism; inflammatory process or benign, tumor-like process suggested	Radiographic or histopathological diagnosis	Peak of disease onset age 20–40 years, pain/swelling of joints (mainly knee or hip, rarely temporomandibular joint) with “locking phenomenon”, fatigue	Elevated ESR and CRP possible	Surgical synovectomy, radiotherapy, immunotherapy	Chronic or remitting-relapsing, locally aggressive and frequent relapses	Arthritis/ arthropathy
Felty syndrome (splenomegaly-neutropenia-rheumatoid arthritis syndrome) [119–122]	1% of RA = estimated prevalence 1/10,000	HLA-DR4 association (78%), autoantibodies against neutrophil extracellular chromatin traps (NETs), anti-GCSF antibodies	Clinical diagnosis	More common in females, chronic symmetric arthritis with severe joint destruction (often knee, wrist ankle, MCP, PIP), hepatosplenomegaly, lymphadenopathy, episcleritis, pleuritis, vasculitis, weight loss	Anemia, neutropenia, infections, ANA, RF	Corticosteroids, DMARDs, biologicals, G-CSF, splenectomy	Chronic, increased mortality due to infections	Arthritis/ arthropathy
RS3PE (remitting seronegative symmetrical synovitis with pitting edema) [3, 32, 123, 124]	Unknown, prevalence in Japan 0.09% (90/100,000 = 1/1,111) for > 50 years (not rare among elderly), estimate 1/100,000	Unknown; associated with other rheumatic conditions, infections, and neoplasms (associated malignancy rate 7% in Asia and 31% in Western countries); VEGF may play a role in pathogenesis	Proposed diagnostic criteria by Karmacharya et al.	Usually older males affected, symmetrical synovitis of hands and ankles, sudden onset polyarthritis, pitting edema (especially hands or feet)	Elevated acute phase reactants, usually RF and autoantibodies negative	Usually excellent response to corticosteroids, DMARDs rarely used, treatment of underlying malignancy if present	Good, remission can usually be achieved with corticosteroid use only, poor prognosis if malignancy-associated	Arthritis/ arthropathy
Multicentric reticulohistiocytosis [37, 125–127]	Unknown, ~ 300 cases in literature, estimate < 1/1,000,000	Unknown; different triggers suspected (malignancies, autoimmune diseases, infections/tuberculosis); crucial workup for neoplasms necessary; macrophages, cytokines, and osteoclastic activity seem to play a role in pathogenesis	Histopathological diagnosis	Mainly Caucasian females (3:1) with peak of onset in 4th decade, symmetric erosive polyarthritis or spondylitis with axial involvement (most affected joints: distal interphalangeal joints) with typical papulonodular skin lesions, organ involvement possible (heart, lung), arthritis often precedes skin involvement by years	Elevated ESR, CRP, anemia, hyperlipidemia, different autoantibodies may be positive	NSAIDs, corticosteroids, DMARDs, biologicals, bisphosphonates, treatment of underlying malignancy if present	Variable, may progress rapidly into arthritis mutilans, but most patients achieve remission spontaneously within 10 years	Arthritis/ arthropathy
Chronic non-bacterial osteomyelitis (CNO)/CRMO/SAPHO [128–132]	Estimated prevalence 40/100,000	No clear association with HLA-B27, possibly related to other genes connected to autoinflammatory disorders; autoimmune process or infection/molecular mimicry (*P. acnes*) suspected	Inclusion and exclusion criteria by Benhamou et al., Kahn et al.	Slight female predominance, onset often in children or middle-aged adults; inflammatory, painful, sterile (sometimes *P. acnes*-positive) osteitis (often in anterior chest wall or axial skeleton) with variety of different skin diseases (most commonly palmoplantar pustulosis), onset can be many years before or after bone and articular involvement (often sacroiliac or sternoclavicular joints)	Elevated CRP and ESR (sometimes), different non-specific autoantibodies, sometimes *P. acnes*	NSAIDs, corticosteroids, bisphosphonates, antimicrobial treatment, DMARDs, biologicals	Chronic disease, complications or disease-associated death rare	Arthritis/ arthropathy
Systemic-onset juvenile idiopathic arthritis (Still`s disease) [133–141]	Estimated prevalence 10.5/100,000	Association with *MEFV* and MIF-173 polymorphism; IL-6 and IL-1 play a major role	ILAR classification criteria for JIA	Systemic inflammation (spiking fever > 39 °C, skin rash, hepatosplenomegaly, lymphadenopathy, serositis, arthritis	High levels of serum ferritin, marked polymorphonuclear leukocytosis, thrombocytosis, elevated ESR/CRP	NSAIDs, corticosteroids, DMARDs, biologicals (IL-1, IL-6)	Variable, chronic, self-limiting or remitting-relapsing, ~ 50% complete recovery, risk of MAS	Arthritis/ arthropathy
Whipple's disease [142–149]	Unknown, *Tropheryma whipplei* can be found in ~ 10% (Europe)/20% (Africa) of fecal samples in healthy population, estimate < 1/1,000,000	HLA-B27 involvement discussed; infection with *Tropheryma whipplei*, predominantly male patients and patients with immune modulatory conditions (alcohol, abuse, chronic disease) affected	Histopathological diagnosis or PCR	Predominantly males affected, intermittent polyarthritis and gastrointestinal symptoms, any other organ can be affected (neurology, cardiovascular, lungs, eyes, skin), fever, weight loss, abdominal pain, malabsorption, headaches, diarrhea	Elevated ESR, CRP, anemia, thrombocytosis, reduced IgM and IgA, leukocytosis, RF, anti-CCP-AB may be present	Antibiotics, corticosteroids, DMARDs, biologicals (IL-1)	Chronic, lethal if untreated, increased mortality in case of neurological involvement or occurrence of immune reconstitution inflammatory syndrome (IRIS; ~ 10%)	Arthritis/ arthropathy
Osteochondritis dissecans [150–152]	Incidence 6.09/100,000 Estimated 15–29/100,000 → 22/100,000	Unknown; osteonecrosis of subchondral bone; vascular disruption, multiple microtrauma, and genetic predisposition suggested	Radiographic diagnosis	Predominantly in physically active male children or adolescents. Pain (worsening with exercise), swelling, joint locking, most often in the knee, but any joint can be affected	Usually inflammatory markers normal	Supportive: NSAIDs, surgery	Chronic, prognosis depends on stability of lesion and patient age	Arthritis/ arthropathy
Blau syndrome (familial)/early onset sarcoidosis (sporadic)/ pediatric granulomatous arthritis [9, 153–158]	Unknown, incidence: 0.29/100,000 (Denmark), estimate < 1/1,000,000	Autosomal dominant, sporadic form, gonosomal mosaicism in *NOD2/CARD15*; NF-κB activation and excessive inflammatory cytokine production	Clinical diagnosis, genetic testing, skin biopsy	Skin rash in first year of life, later boggy polyarthritis, uveitis, non-caseating epithelioid and giant cell granulomas; fever, lymphadenopathy, neuropathy, renal/hepatic/lung/cardiovascular involvement	Leukocytosis, thrombocytosis, elevated ESR, CRP, acute-phase reactants, ACE normal	NSAIDs, corticosteroids, immunosuppressants, biologicals, systemic hypertension usually responds to ACE-inhibitors	Chronic, prognosis depends on systemic involvement, severe ocular and articular morbidity	Autoinflammatory syndrome
CAPS (familial cold autoinflammatory syndrome/familial cold urticaria, Muckle-Wells syndrome, CINCA syndrome/NOMID/IOMID) [38, 39, 159–161]	1–2/1,000,000 in US and 1–2/360,000 (= 4.6/1,000,000) in France estimated → 3.05/1,000,000	Autosomal dominant, gain of function mutation in *NLRP3/CIAS1* leads to caspase-1 and inflammasome activation with increased IL-1β secretion; mosaicism possible, usually de novo	Eurofever clinical diagnostic/ classification	Intermittent fever, urticarial rash, chronic inflammation, typical facies in CINCA (frontal bossing, saddle back nose), CNS manifestations, chronic polyarthritis, conjunctivitis, papilledema	Elevation of acute phase reactants, leukocytosis, chronic anemia; SAA biomarker for development of AA-amyloidosis	Biologicals (IL-1)	Chronic, prognosis significantly improved since availability of anti-IL-1-treatment (65–85% complete remission with Anakinra)	Autoinflammatory syndrome
Familial Mediterranean fever (familial paroxysmal polyserositis) [38, 162–166]	Prevalence highly differs geographically; in eastern Mediterranean 1/500 – 1/1000, Turkey 1/150 – 1/10,000, Ashkenazi Jews 1/73,000 Estimate 1/10,000	Autosomal recessive, mutation in *MEFV* leading to abnormal function of inflammasome; environmental factors also seem to play a role, as patients from the eastern Mediterranean often have a milder phenotype	Multiple criteria: Eurofever clinical diagnostic/classification criteria, Tel-Hashomer, Yalcinkaya-Ozen and Livneh-criteria	Disease onset usually in childhood, 90% before age of 20 years, recurrent fever and serositis, myalgia, arthralgia, abdominal pain, vomiting, chest pain, rash, prodromal phase with unspecific symptoms (restlessness, anxiety, irritability), rapid onset of symptoms lasting for at least 12 h	Elevated acute phase reactants	Colchicine, anti-IL-1	Chronic, remission and fewer relapses can be achieved by therapy, complications include amyloidosis (strongest predictor seems to be country of residence) and MAS	Autoinflammatory syndrome
Mevalonate kinase deficiency (hyperimmunoglobulinemia D with periodic fever, HIDS) [38, 167–170]	Unknown, incidence in Germany: 0.39/1,000,000 estimate < 1/1,000,000	Autosomal recessive, mutations in *MVK* (homozygosity or, most often, compound heterozygosity); MVK essential for cholesterol synthesis; increased production of IL-1β; possible founder effect in the Netherlands	Eurofever clinical diagnostic/classification criteria	Recurrent fever episodes starting in infancy (most common before end of 1st year of life), fever lasts 4–6 days and can be provoked by physical and psychological stress; lymphadenopathy, splenomegaly, arthralgia, GI symptoms, skin rash, sometimes oral and vaginal aphthous ulcers, neurological symptoms	Elevated ESR, CRP, leukocytosis, elevated IgD (> 100 IU/ml), IgA in blood, elevated mevalonic acid in urine	HMG-CoA-reductase inhibitors, corticosteroids, immunosuppressants, biologicals (Il-1)	Chronic, complications include infections, amyloidosis, peritonitis with abdominal adhesions, MAS, and joint contractures	Autoinflammatory syndrome
Nakajo-Nishimura syndrome (NNS) [171–173]	Unknown, 28 reported cases in Japan until 2010 (19 males, 9 females), estimate < 1/1,000,000	Autosomal recessive, mutation in *PSMB8*; probably common founder; reduced proteasome activity and accumulation of ubiquitinated and oxidized proteins, leading to increased cytokine levels	Distinctive clinical diagnostic criteria for NNS	Onset usually at age of 2 months—8 years with pernio-like rash, rash often appears in first winter after birth and reappears every year. Symptoms worsen with cold stimuli; periodic high fever, skin rash, myositis, hepatosplenomegaly, partial lipomuscular atrophy, joint contracture (mainly in upper body), hyperhidrosis, short stature, low IQ, lymphadenopathy described, characteristic thin facial appearance, elongated clubbed fingers	Constantly elevated ESR, CRP, chronic anemia, hypergammaglobulinemia, elevated IgG and IgE, positive ANA described	Corticosteroids, kallikrein, dapsone	Chronic and often lethal, most patients die of cardiac or respiratory failure	Autoinflammatory syndrome
PAPA syndrome (pyogenic arthritis-pyoderma gangrenosum acne syndrome) [28, 39, 174–177]	Only few patients from five families worldwide reported (34 until 2006), estimate < 1/1,000,000	Autosomal dominant, missense mutation in *PSTPIP1/ CD2BP1*, which causes hyperphosphorylation of PSTPIP1 protein and induction of inflammasome	Clinical diagnosis with genetic confirmation	Recurrent sterile arthritis, fever, pustulosis, abdominal pain, hidradenitis, acne, pyoderma gangrenosum, ulcerations	Elevated ESR, CRP, IL-1β	Corticosteroids, biologicals (IL-1 β)	Chronic or remitting-relapsing	Autoinflammatory syndrome
PFAPA syndrome (periodic fever, aphthous stomatitis, pharyngitis, cervical adenitis; Marshall syndrome with periodic fever) [178–182]	Unknown, incidence 2.3/10,000 in children up to 5 years (Norway), probably not so rare, estimate < 1/10,000	Unknown; many patients have heterozygous *MEFV* mutation, familial occurrence; polygenetic cause suspected; IL-1 and vitamin D may play a role in pathogenesis	Diagnostic criteria by Marshall/Thomas; Cantarini et al.	Disease onset usually in early infancy, slight male predominance, episodes last 5 days and recur every 28 days; prodromal: aphthous stomatitis, malaise, fatigue, irritability, headache; then sudden onset of high fever, pharyngitis, lymphadenopathy, chills, cough, headache, abdominal pain, nausea, diarrhea, rash; patients are remarkably healthy between episodes	Leukocytosis and elevated ESR in episodes	Corticosteroids, surgery (tonsillectomy), cimetidine, anakinra, colchicine	Good prognosis, self-limited within 4–5 years, normal development	Autoinflammatory syndrome
Schnitzler syndrome (chronic urticaria with gammopathy) [183–186]	Unknown, ~ 250 reported patients, mainly from western Europe, estimate < 1/1,000,000	Unknown; involvement of IL-1β and IL-6 suspected	Strasbourg diagnostic criteria	Slight female predominance (1.6:1), disease onset in adulthood (mean age 51 years), recurrent urticarial rash (most constant symptom), fever, muscle/bone/joint pain, lymphadenopathy	Monoclonal IgM (rarely IgG) gammopathy, elevated ESR, κ -light chain, leukocytosis	Anakinra (IL-1) rapidly controls symptoms (diagnosis should be reconsidered if ineffective), canakinumab, colchicine, NSAIDs, pefloxacin, hydroxychloroquine	Chronic, spontaneous remission and relapses common, complications include amyloidosis and overt lymphoproliferation	Autoinflammatory syndrome
Macrophage activation syndrome [187–192]	Seen in about 10% of patients with systemic onset JIA. Prevalence unknown, estimate < 1/1,000,000	Excessive Activation of T-lymphocytes and macrophages. Possible association with impaired NK cell cytotoxicity due to *PRF1* mutation	HLH-2004 diagnostic guidelines/2016 criteria for MAS complicating systemic JIA	Fever, hepatosplenomegaly, cytopenias, coagulopathy, liver dysfunction, neurological symptoms, lymphadenopathy, skin rash, jaundice, edema	cytopenia, elevated transaminases + ferritine, low NK cell activity, elevates sIL2-R	Corticosteroids, Cyclosporine, Biologicals, IL-1 receptor blockade	Mortality in one retrospective study 8% (higher mortality in adults)	Autoinflammatory syndrome
Majeed syndrome (chronic recurrent multifocal osteomyelitis) [193–195]	Unknown, only 4 families/14 patients with Middle Eastern origin reported, estimate < 1/1,000,000	Autosomal recessive, mutation in *LPIN2*, which plays a role in fat metabolism and, possibly, mitosis	Clinical diagnosis, genetic testing	Inflammation of bone and skin, resulting in growth disturbances and joint contractures; recurrent high fevers, severe pain; frequently associated with cutaneous inflammatory syndromes (e.g., psoriasis, Sweet syndrome)	Dyserythropoietic anemia with microcytosis, elevated ESR	Blood transfusions, NSAIDs, corticosteroids, biologicals (IL1 β)	Chronic, osteomyelitis probably life-long, anemia is prominent in childhood	Autoinflammatory syndrome
TRAPS (tumor necrosis factor receptor 1 associated periodic syndrome; familial Hibernian fever) [38, 39, 196–198]	Unknown, incidence 1/1,785,714 for children < 16 (Germany); most patients are European Caucasian, estimate 1/1,000,000	Autosomal dominant with variable penetrance, mutations in *TNFRSF1A*, different hypotheses on pathophysiology, including intracellular trafficking, receptor shedding, or induction of apoptosis, leading to increase in cytokines; triggers include stress, menstrual cycle, fatigue, infections, exercise, vaccinations	Eurofever clinical diagnostic/ classification criteria	Disease onset usually in infancy or childhood, attacks last around 11 days, on average 70 symptomatic days a year with high fever, limb pain, abdominal pain, rash, cervical lymphadenopathy, periorbital edema	Elevated ESR, CRP, leukocytosis, thrombocytosis, anemia, hypergammaglobulinemia; SAA levels correlate with disease activity	NSAIDS, corticosteroids, biologicals (most promising is anti-IL-1)	Often remitting-relapsing, but chronic course possible; complications include amyloidosis and MAS	Autoinflammatory syndrome
Necrotizing autoimmune myopathy (anti-HMG-CoA myopathy) [199, 200]	Unknown, estimate < 1/1,000,000	Unknown; immune-mediated muscle fiber necrosis without inflammation due to statin use, other drugs, malignancies, or connective tissue diseases	Histopathological diagnosis	Female predominance (73%), myalgia, dysphagia, weight loss, fatigue, ILD, arthralgia, Raynaud´s phenomenon	Anti-SRP antibodies present in 16%, anti-HMGCR antibodies seem to be specific and present in ~ 60% of patients previously exposed to statins; CRP and CK elevated	Statin withdrawal, corticosteroids, DMARDs	Variable, good prognosis in case of treatable underlying cause, but chronic in most cases	Myositis/myopathy
Antisynthetase syndrome [201–204]	Unknown, 20–25% of patients diagnosed with PM/DM; prevalence of PM/DM approx. 15/100,000 → 3.4/100,000	Unknown; antibodies against anti-threonyl-tRNA-synthetase; relationship to exposure to airborne particles discussed	Clinical diagnosis + antibody findings	Females 2–3 times more often affected than males, at presentation often only RA-like arthritis, then inflammatory myopathy, interstitial lung disease, fever, Raynaud´s, Gottron´s papules, mechanic´s hands	Anti-Jo-1, anti-Pl7/PL12, many other antibodies possibly positive	Corticosteroids, immunosuppressants (rituximab), DMARDs	Chronic, overall survival good but decreased in case of lung involvement	Myositis/myopathy
Myopathic form of carnitine palmitoyltransferase II (CPT II) deficiency [205, 206]	Unknown, more than 300 published cases, one of the most common disorders of oxidative fatty acid metabolism, prevalence probably higher than suspected; estimate < 1/100,000	Autosomal recessive, mutation in *CPTII*; CPT is involved in the transportation of long chain fatty acids in mitochondria; impaired energy metabolism; frequent triggers are physical stress and exposure to cold	Enzyme measurement, genetic testing	Disease onset in adolescence or adulthood, males more commonly and more severely affected; myalgia, rhabdomyolysis, muscle weakness, pain, lipid accumulation in muscle	Elevated CK, BUN, myoglobinuria, hepatic steatosis	Avoidance of triggers (fasting, prolonged exercise), low fat and high carbohydrate diet, carnitine	Chronic, but good prognosis; rhabdomyolysis can lead to renal failure	Myositis/myopathy
Dermatomyositis/Polymyositis [207–210]	10–13/100,000 (Japan) 8.7/100,000 (Norway) 7–10/100,000 (Brazil) → 9.6/100,000	Unknown; humoral-mediated inflammation in DM, cell-mediated (CD8 + T-cells) in PM; often associated with other autoimmune diseases and malignancies	Histopathological diagnosis	Predominantly females, myalgia, arthritis, dyspnea, dysphagia, muscle weakness, rash (not in PM), myocarditis, Gottron´s papules	Different myositis-specific autoantibodies can be found: anti-Jo-1, NXP2/MJ antibody, anti-155/140 antibodies, anti-MDA5, MI-2-antibodies	Corticosteroids, immunosuppressants	Variable, most patients improve over time with treatment, prognosis depends on associated diseases (malignancies)	Myositis/myopathy
Inclusion body myositis [31, 211, 212]	33/1,000,000 (Norway) 10.7 – 71 /1,000,000 (USA) → 3.7/100,000	Hereditary autosomal recessive form with onset in young adults, mutations in *GNE* (very rare); sporadic form in elderly, associated with HLA-DR3 and MHC variants; genetic, environmental, aging, and immune-mediated factors probably related to pathogenesis	Histopathological diagnosis	Predominantly males (2:1) and elderly, slowly progressive muscle weakness (often beginning in wrists or quadriceps muscle), dysphagia	CK may be elevated or normal	Refractory to immune therapy, can be used tentatively in case of relation to other autoimmune diseases	Chronic and slowly progressing, most patients wheelchair-reliant within 10 years	Myositis/myopathy
Eosinophilia-myalgia syndrome [213]	Unknown, 5000–10,000 people affected, predominantly females in the US (epidemic in 1989), estimate < 1/1,000,000	Unknown; consumption of manufactured L-tryptophan or 5-hydroxytryptophan associated with disease onset; increased TGF-β and IL-4 may be responsible for fibrosis	Clinical diagnosis	Rapid onset of severe myalgia, cough, fever, fatigue, joint pain, edema; long-term symptoms include eosinophilic fasciitis, alopecia, CNS involvement, myocarditis, arrhythmias, GI involvement	Elevation of eosinophils, WBC	Supportive treatment, corticosteroids in acute phase may be used tentatively	Chronic course with systemic organ involvement not uncommon	Myositis/ myopathy
Focal myositis [214, 215]	Unknown, estimate < 1/1,000,000	Unknown; trigger factors poorly understood (e.g., physical trauma)	Clinical, radiographic, and histopathological diagnosis	Rapidly growing mass in a single muscle, most commonly in lower limbs, usually painless	Usually no elevated acute phase reactants, CK may be elevated but usually normal	No treatment, corticosteroids in case of inflammation or complications	Usually self-limited within few weeks or months, relapses possible but uncommon	Myositis/myopathy
McArdle’s disease (glycogenosis type 5) [216–219]	Estimated 1:50,000 (US) – 1:140,000 (Spain) → 1.4/100,000	Autosomal recessive, mutations in *PGYM*, leading to glycogen phosphorylase deficiency	Enzyme measurement, histopathology, genetic testing	High clinical variability, rapid fatigue, myalgia and cramping with exercise and fast recovering with rest (“second-wind phenomenon”)	Elevated baseline CK, myoglobinuria, rhabdomyolysis	No treatment; moderately active lifestyle and ingestion of simple carbohydrates before exercise recommended	Chronic, but variable in severity; complications include renal failure and cardiovascular diseases	Myositis/myopathy
Tarui disease (GSD7) [220, 221]	Unknown, more than 100 cases described, Common in Ashkenazi-Jews, estimate < 1/1,000,000	Autosomal recessive, mutations in *PFKM*, leading to muscle phosphofructokinase deficiency	Histopathological diagnosis, genetic testing	Exercise intolerance, myalgia, cramps, cardiomyopathy	Myoglobinuria, hemolytic anemia, hyperuricemia, hyperCKemia, reticulocytosis	No treatment; avoid extensive exercise	Chronic, complications include renal and cardiac involvement	Myositis/myopathy
Camurati-Engelmann disease (progressive diaphyseal dysplasia) [26, 222, 223]	Unknown, estimate < 1/1,000,000	Autosomal dominant, mutations in *TGFB1*, resulting in increased growth factor signaling	Genetic testing, clinical findings + radiographic images	Hyperostosis of long bones and skull, severe limb pain, muscle atrophy, wide-based waddling gait, progressive joint contractures, hearing loss, absence of subcutaneous fat	Increased levels of TGF-β1	Corticosteroids, NSAIDs, bisphosphonates, all with variable outcomes; experimental: anti-TGFβ (e.g., losartan)	Chronic, patients may become wheelchair-reliant	Bone disorder
Fibrodysplasia ossificans progressiva (Munchmeyer's disease) [224, 225]	0.36/1,000,000 (Spain) 1.36/1,000,000 (France) estimated worldwide prevalence (literature): 1/2,000,000 → 0.74/1,000,000	Autosomal dominant (most cases de novo), mutation in *ACVR1*, leading to enhanced BMP signaling with fibroproliferation, angiogenesis, enchondral ossification; risk seems to be increased with older age of mother and father, fathers often exposed to chemicals	Clinical, radiographic, histopathological diagnosis	Heterotopic ossification, tumor-like swellings and short, malformed great toes (early sign), cervical spine fusions, osteochondroma, hearing loss	Usually normal, although ESR and AP may be elevated	Short-term muscle relaxants, NSAIDs, Cox-2-inhibitors, corticosteroids, bisphosphonates; operations should be avoided (triggers new flare ups and bone growth)	Chronic, progressive, and lethal within approximately 40 years, most patients wheelchair-reliant at the end of second decade of life	Bone disorder
Osteomesopyknosis [226]	Unknown, < 50 cases reported, predominantly from France, estimate < 1/1,000,000	Autosomal dominant, gene unknown	Radiographic diagnosis	Disease onset and diagnosis usually in adolescence, diffuse back pain	Usually normal	Symptomatic	Benign and good prognosis, normal life expectancy	Bone disorder
Fabry disease [227–229]	Australia 0,85/100,000	alpha-galactosidase A deficiency due to mutation in *GLA*-gene on X-chromosome (X-linked disorder)	Measurement of enzyme activity, genetic testing	Neuropathic pain, hypohidrosis, gastrointestinal symptoms, kidney failure, cardiovascular disease	renal function may be impaired	Enzyme replacement therapy	Chronic. Life expectancy increased with ERT, but limited by cardiovascular and renal function	Bone disorder
Farber disease [230, 231]	Unknown, estimate < 1/1,000,000	Autosomal-recessive, *ASAH1*-gene; acid ceramidase deficiency	Measurement of enzyme activity; histopathology of granuloma; ceramide accumulation in granuloma	Subcutaneous nodules, joint disease, hoarseness of voice, inflammatory granuloma		Enzyme replacement therapy in progress; Stem cell transplantation	Chronic and progressive. Death due to respiratory insufficiency caused by pulmonary granulomas	Bone disorder
Gaucher's disease (type 1 in 90% of cases) [232–235]	Estimated 1–2/100,000 worldwide, 1/850 in Ashkenazi-Jews → 1.5/100,000	Autosomal recessive, mutations in *GBA1*; deficiency in lysosomal glucocerebrosidase leads to accumulation of glucocerebroside	Measurement of enzyme activity, genetic testing	Age of onset and disease course variable; fatigue, growth retardation, delayed puberty, bone pain, avascular necrosis of bone, gallstones, hepatosplenomegaly, Parkinson´s disease, malignancies (predominantly hematological)	Thrombocytopenia, anemia, monoclonal gammopathy, vitamin D deficiency; biomarkers: chitotriosidase, CCL 18, glucosylsphingosine, ferritine	Lifelong enzyme replacement or substrate reduction therapy	Chronic, reduced life expectancy due to neurological involvement and malignancies	Bone disorder
Hypophosphatasia (HP) [236–238]	1/300.000 for severe HP, 1/6370 for moderate HP Estimate 1/100,000	Autosomal-recessive or autosomal-dominant; mutations in *TNSALP* lead to accumulation of pyrophosphate, an inhibitor of mineralization	Laboratory values + radiologic features, confirmed by genetic testing	Age of onset and disease course very variable; perinatal death, bone deformities, stress fractures, loss of dentition, musculoskeletal pain and weakness	low serum AP, hypercalcemia	Enzyme replacement therapy for pediatric onset hypophosphatasia	Chronic, mortality and morbidity varies with onset	Bone disorder
Morquio disease (mucopolysaccharidosis type IVa) [239–241]	1/323,000 (Denmark) 1/599,000 (UK) 1/1,872,000 (Malaysia) 1/926,000 (Australia) → 1.6/1,000,000	Autosomal recessive, mutations in *GALNS*, resulting in N-acetylgalactosamine-6-sulfate sulfatase-deficiency	Measurement of enzyme activity, genetic testing	Disease onset in childhood; progressive skeletal dysplasia, short trunk dwarfism, spondyloepiphyseal dysplasia with ligamentous laxity, joint pain, preserved intelligence, odontoid hypoplasia pulmonary, cardiac, ophthalmologic, audiologic, dental, abdominal and neurologic involvement possible	GAGs in urine	Enzyme replacement therapy, pain management, supportive therapy, surgery	Chronic, wheelchair-reliance beginning in adolescence, increased mortality due to cervical instability and pulmonary compromise	Bone disorder
Melorheostosis (Leri´s disease) [105, 242–244]	~ 400 cases reported, estimate 1/1,000,000	Usually sporadic; somatic *LEMD3* mutations suspected as a possible cause; disturbance in bone formation and modeling; possible association with scleroderma and Buschke-Ollendorf syndrome	Radiographic diagnostic criteria (Freyschmidt)	Disease onset in childhood or adolescence; limb deformity, contractures, joint and bone pain, leg length discrepancy, stiffness, hyperostosis (usually long bones in lower extremity) usually in one limb, but may be bilateral, soft tissue involvement (hypertrichosis, fibrosis, erythema) above affected bone	Markers of bone metabolism usually normal (calcium, AP, etc.)	Pain management, bisphosphonates, surgery (relapses common)	Chronic and progressive, morbidity mostly due to pain, stiffness, and reduced range of motion	Bone disorder
Pachydermoperiostosis (primary hypertrophic osteoarthropathy; Touraine-Solente-Golé syndrome) [23, 24, 245–247]	Unknown, estimate < 1/1,000,000	Autosomal dominant with incomplete penetrance proven, autosomal recessive and X-linked inheritance also suggested, mutations in *SLCOA21*, *HPGD*, possibly also related to HLA-B12 or BMP pathway; involvement of testosterone promoting proliferation suspected	Clinical diagnosis	Occurs predominantly in males (7:1), disease onset often in puberty, progression for 5–20 years; pachydermia, digital clubbing, periostosis, cranioosteoarthropathy, congenital heart diseases (especially patent ductus arteriosus), hyperhidrosis, rash, myelofibrosis, gastrointestinal involvement	Unspecific	NSAIDs, corticosteroids, colchicine, bisphosphonates, retinoids, plastic surgery	Chronic, progressive for 5–20 years	Bone disorder
Mucopolysaccharidosis type 2 (Hunter syndrome) [248–250]	0.65/1,000,000 (Sweden) 0.44/1,000,000 (Norway) 0.91/1,000,000 (Denmark) → 0.67/1,000,000	X-linked recessive, mutation in *IDS*; lysosomal storage disorder: iduronate-2-sulfatase enzyme deficiency	Measurement of enzyme activity, genetic testing	Disease onset in childhood; peau d´orange, cognitive impairment, joint stiffness, contractures, cardiac and respiratory involvement, short stature, carpal tunnel syndrome, hepatosplenomegaly, glaucoma	GAGs in urine	Enzyme replacement therapy, supportive treatment, pain management	Chronic, often lethal within 20—30 years (cardiovascular involvement limiting), patients with attenuated form may have normal life expectancy	Bone disorder
Caffey disease (infantile cortical hyperostosis, Caffey-Silverman syndrome, Smyth syndrome) [251–253]	Unknown, estimate < 1/1,000,000	Autosomal dominant, heterozygous mutation in *COL1A1* with incomplete penetrance	Clinical and radiographic diagnosis with genetic confirmation	Fever, swelling of soft tissues, hyperostosis of outer cortical surface in first 5 months of life, unusual irritability	Elevated ESR, AP, thrombocytosis, anemia, increased immunoglobulin	Symptomatic: NSAIDs	Good, usually self-limiting in early childhood, chronic or remitting-relapsing course possible	Connective tissue disease
Ehlers-Danlos syndrome [254–256]	1/10,000 – 1/25,000 = 7/100,000	Autosomal dominant or autosomal recessive, mutations in *COL5A1/COL5A2/COL5A3/COL3A1* and other, depending on subtype	Villefranche classification	Depending on subtype: joint hyperlaxity and luxation, easy bruising, arthralgia, vascular aneurysm, muscle hypotonia	Normal coagulation status despite easy bruising	Only symptomatic and supportive treatment	Chronic, worst prognosis in vascular subtype, obstetrical complications common	Connective tissue disease
Fibrosing mediastinitis [257–259]	Unknown, estimate < 1/1,000,000	Most cases idiopathic, or due to infections (histoplasma, aspergillus) or sarcoidosis; proliferation of fibrous tissue, possibly IgG4-related	Radiographic diagnosis	Often younger people affected; cough, hemoptysis, dyspnea, other symptoms depend on grade of obstruction of surrounding structures	Usually normal	Corticosteroids, local therapies, surgery	Variable, often chronic and progressive, potentially lethal because of invading/ displacing growth	Connective tissue disease
IgG4-related Disease [260–263]	6/100.000 (Japan)	Immune mediated, multiple possible risk factors	ACR/EULAR Classification Criteria	Elderly men primarily affected; any organ involvement possible, most often gastrointestinal organs or salivary glands, leading to fibrosis and subsequent organ dysfunction	Serum IgG4 may be elevated	Corticosteroids	Chronic, remitting-relapsing. Usually mild symptoms, only slowly progressing	Connective tissue disease
Marfan syndrome [264–266]	6.5/100,000 (genetically proven patients in Denmark)	Autosomal dominant, mutation in *FBN1*, resulting in disturbed fibrillin 1 function and altered TGF β regulation, large phenotypic variability	Revised Ghent criteria	Tall stature, joint hypermobility, arachnodactyly, aortic aneurysm, mitral valve prolapse, ectopia lentis, scoliosis, dural ectasia	Usually normal	β–blockers, cardiac and/or orthopedic surgery	Chronic, mortality depends on cardiovascular involvement, life expectancy normal with regular follow-up and treatment	Connective tissue disease
Shulman disease (eosinophilic fasciitis) [267, 268]	Unknown, estimate < 1/1,000,000	Association with *HLA-A2* described, many different theories on pathogenesis and possible triggers (e.g., physical exercise) exist	Proposed diagnostic criteria by Pinal-Fernandez et al.	Disease onset at any age (mean 4th-5th decade); abrupt onset of painful swelling and thickening of skin and other soft tissues, joint contractures, most often extremities symmetrical involved	Blood eosinophilia, hypergammaglobulinemia, elevated ESR, TIMP-1 possibly marker for disease activity	Corticosteroids, MTX; not all patients require treatment	Variable, remission usually occurs spontaneously or with therapy	Connective tissue disease
Sharp syndrome (mixed connective tissue disease) [269–271]	3.8/100,000	Unknown, B cells may play a role in pathogenesis	Different diagnostic criteria exist (Sharp´s, Alarcón-Segovia and Villareal, Kasukawa)	Female predominance (3.3:1), Raynaud´s phenomenon, puffy hands, arthritis, pleuritis, pericarditis, myositis, interstitial lung disease, PAH, esophageal dysmotility, dyspnea, cardiovascular involvement	Anti-ribonucleoprotein antibodies (anti-U1RNP)	NSAIDs, corticosteroids, immunosuppressants	Chronic and progressive, may evolve into other connective tissue disease, mortality increased with cardiovascular involvement	Connective tissue disease
Systemic sclerosis [30, 44, 45, 272–275]	More common in Europe than Asia, less common in northern countries, highest ever reported prevalence in population of Choctaw Indians in Oklahoma (469/100,000), worldwide → 15–30/100,000 (= 22.5)	Unknown, HLA-association suspected, different pathophysiological factors suspected (vasculopathy, autoantibodies, fibroblast dysfunction, immune system alteration, silica dust, toxins, infections)	ACR criteria	Female predominance (3:1), peak incidence at age 45 – 64 years; skin thickening, Raynaud´s phenomenon, pulmonary fibrosis, PAH, digital ulcers, esophageal hypomobility, arthralgia, myalgia, variable organ involvement	Anti-centromere-AB, anti-topoisomerase-I-AB (Scl70), anti-RNA-polymerase III	Symptomatic and supportive treatment of Raynaud´s phenomenon, digital ulcer, skin, lung, and GI disease	Chronic and progressive, worst prognosis among all connective tissue diseases, mean survival 11–12 years after diagnosis	Connective tissue disease
CLOVES syndrome (congenital lipomatous overgrowth, vascular malformations, epidermal nevi syndrome) [276–279]	Unknown, only a few cases reported, estimate < 1/1,000,000	Mosaic activating, postzygotic mutation in *PIK3CA*, causing tissue overgrowth	Clinical diagnostic criteria for PIK3CA-related overgrowth spectrum (Keppler-Noreuil et al.), genetic testing	Vascular malformations, thoracic lipomatous hyperplasia, asymmetric growth, visceral and neurological disorders, linear epidermal nevus, gigantism of hand and feet, macrodactyly, sandal gap toe, renal anomalies	Normal	Clinical trials with mTOR kinase-inhibitors and selective PIK3CA-inhibitors; laser, sclerosing, or surgical treatment	Chronic, severity depends on somatic mosaic, frequent recurrence of lipomatous masses, increased risk of tumors (e.g., Wilms tumor)	Overgrowth syndrome
Klippel-Trénaunay-Weber syndrome complex (angio-osteohypertrophic syndrome = Klippel-Trenaunay, special form with AV fistulas = Parkes-Weber syndrome) [280–283]	Unknown, ~ 1000 reported cases in literature, estimated incidence 1/100,000 estimate < 1/1,000,000	Unknown, multiple different inheritance modes suspected; current candidate genes: *VG5Q*, *PIK3CA*, *AGGF1*, *ING5*, *HDAC9*; congenital defects in spinal cord, vessels, and mesodermal tissues suspected	Clinical and radiographic diagnosis	Cutaneous capillary malformations (portwine stain), varicous veins, hypertrophy of bone and soft tissue (often resulting in different limb lengths), usually isolated to one extremity (most commonly leg), pain, edema, pruritus; in Parkes-Weber syndrome: + AV fistulas	Normal	Symptomatic: compression stockings, laser surgery, treatment of infections, thromboembolic events	Chronic, but rarely cause of death, higher mortality in Parkes-Weber syndrome because of AV fistulas, complications include coagulopathy and thromboembolic events	Overgrowth syndrome
Proteus syndrome [284–286]	Unknown, possibly over-/misdiagnosed because of similarities to other overgrowth spectrum disorders, incidence estimated 1/1,000,000 estimate < 1/1,000,000	Somatic mosaic activating *AKT1* mutation, increased growth in affected cells	Revised diagnostic criteria (Turner et al., Cohen), genetic testing of affected tissue	Males more commonly affected than females (2:1); overgrowth of different tissues: cerebriform connective tissue nevus, vascular malformations, deep vein thromboses, dysregulated adipose tissue (lipomas), pulmonary abnormalities, asymmetric and disproportionate overgrowth, tumors, facial phenotype, intellectual impairment, seizures	Coagulopathy and DVT possible	Supportive: antithrombotic prophylaxis, orthopedic surgeries, psychological support	Chronic, premature death in 20% due to respiratory or neurological involvement	Overgrowth syndrome
Erdheim-Chester disease [287–290]	Unknown, ~ 600 reported cases, estimate < 1/1,000,000	In more than 50% *BRAF*-mutations, non-Langerhans cell-histiocytosis with hyperactivation of cytokines	Radiographic and histopathological diagnosis, genetic testing	Predominantly males in 5th-7th decade of life; bone involvement nearly always present, CNS involvement (diabetes insipidus, visual disturbances, pyramidal/extra-pyramidal syndromes), other organ involvement possible (cardiac/lung/retroperitoneal/cutaneous, etc.)	Elevated ESR, AP, or CRP, signs of pituitary insufficiency	Interferon, corticosteroids, immunosuppressants, biologicals (TNFi, IL-1i), BRAF-inhibitors	Chronic and often lethal, 5-year survival < 70%	Other
Hyaline fibromatous syndrome (infantile systemic hyalinosis/juvenile hyaline fibromatosis) [291–294]	Unknown, ~ 150 cases reported (predominantly from Middle East), estimate < 1/1,000,000	Autosomal recessive, mutations in *CMG2/ANTXR2*-gene, CMG2 is a transmembrane protein that plays a role in capillary morphogenesis (also binds anthrax toxins); higher carrier frequency in Middle Eastern populations suspected	Demonstration of hyaline deposition in dermis, genetic testing	Subcutaneous skin nodules, gingival hypertrophy, joint contractures, hyaline deposition, osteopenia. infections, protein-losing enteropathy; cognitive development normal	No specific findings depending on complications (e.g., diarrhea)	Symptomatic: surgery, D-penicillamine, physiotherapy, NSAIDs, nutritional therapy	Chronic, variable course, but often lethal within first 2 years of life (infantile form), oldest known patient is 58 years old	Other
Sweet syndrome (SS, acute febrile neutrophilic dermatosis) [295–300]	Unknown, estimate < 1/1,000,000	Unknown; classic SS (idiopathic), malignancy-associated, and drug-induced histiocytoid SS; commonly related to inflammatory bowel diseases (especially females with Crohn´s disease)	Classic SS: diagnostic criteria by Su and Liu (modified by van den Driesch); drug-induced SS: diagnostic criteria by Walker and Cohen	Slight female predominance, abrupt onset of fever, peripheral neutrophilia, tender erythematous skin lesions, diffuse neutrophilic dermal infiltrate, arthralgia, malaise, headaches, myalgia	Leukocytosis, elevated ESR, CRP	Corticosteroids, potassium iodide, colchicine, immunosuppressants in relapsing cases, treatment of underlying cause if found	Spontaneous or therapy-induced remission, relapses more common in malignancy-associated SS	Other
Relapsing polychondritis [301–306]	2/100,000 (Hungary) estimated prevalence in literature: 4.5/1,000,000 → 1.2/100,000	Association with HLA-DR4, 30% of all patients have associated autoimmune or hematological disease (MDS); vasculitis of all sized vessels occurs	Michet´s criteria, McAdams´ criteria, Damiani and Levine criteria	Typically onset in middle-aged adults; recurrent inflammation of cartilage, especially ears, nose, respiratory tract; vasculitis of all sized vessels, aortic or mitral valve disease, joints, eyes and skin possible	Elevated CRP, ANCA may be positive	NSAIDs, corticosteroids, dapsone, colchicine, immunosuppressants, biologicals	Chronic, survival rate variable, but recent studies report good survival rates	Other
Cogan syndrome [307–309]	Unknown, over 250 cases reported, estimate < 1/1,000,000	Unknown, autoimmune process suggested (additional autoimmune disease diagnosed in ~ 10% of patients), association with cigarette smoking suspected	Clinical diagnostic criteria for typical and atypical Cogan syndrome	Non-syphilitic interstitial keratitis (or other ocular symptoms, then called atypical Cogan), vestibulo-auditory symptoms, fever, weight loss, arthromyalgia, headache	Anemia, leukocytosis, thrombocytosis, elevated ESR or CRP possible	Corticosteroids, DMARDs, immunosuppressants, biologicals; vestibulo-auditory symptoms often unresponsive to treatment	Chronic or remitting-relapsing, complications include persistent hearing loss and cardiovascular involvement with increased mortality	Other
Weber-Christian panniculitis (relapsing febrile nodular nonsuppurative panniculitis; idiopathic lobular panniculitis) [310–313]	Unknown, only a few cases reported, estimate < 1/1,000,000	Unknown, inflammation and necrosis of subcutaneous adipose tissue, mechanism unclear, probably autoimmune	Histopathological diagnosis	Predominantly middle-aged females affected, recurrent subcutaneous inflammatory painful nodules, fever, malaise, arthralgia, hepatosplenomegaly, anorexia, weight loss, ocular inflammation, lung nodules, systemic organ involvement possible	Elevated ESR, anemia, leukocytosis or leucopenia, hypocomplementemia	Corticosteroids, immunosuppressants, biologicals	Chronic, prognosis variable, poor in case of systemic organ involvement	Other
Systemic mastocytosis (mast cell disease) [314–318]	9.59/100,000 (Denmark, including all systemic subtypes)	Somatic gain of function mutation in *KIT*, KIT is a tyrosine kinase receptor essential for correct mast cell development and function	WHO diagnostic criteria	Abnormal proliferation and accumulation of mast cells cause urticaria pigmentosa, flushing, urticaria, GI symptoms, musculoskeletal pain, headaches, anaphylaxis, weight loss, osteoporosis	Anemia, thrombocytopenia, leukocytosis, eosinophilia, elevated tryptase, uric acid, LDH, bilirubin, ferritin, hypoalbuminemia	Imatinib, symptomatic treatment, interferon-α, corticosteroids, 2-chlorodeoxyadenosine	Chronic, variable progression, may evolve into leukemia	Other
Sarcoidosis (Boeck’s sarcoid) [319–321]	11.16/100,000 (Northern Ireland) 28.13/100,000 (Ireland) → 19.6/100,000	Associated with different *HLA* subtypes and *BTNL-2*, inhaled antigens are considered a possible trigger	Clinical and histopathological diagnosis	Females more often and more severely affected, peak onset in second decade, many patients are asymptomatic or have unspecific symptoms, such as fever, fatigue, weight loss; most commonly affected organ: lung	Elevated acute phase reactants, ACE, s-IL2R	Corticosteroids, immunosuppressants, biologicals	Variable, often self-limiting within 24 months, increased mortality with systemic organ involvement	Other

## Abbreviations

AKT: Serine/threonine kinase 1
ANA: Antinuclear antibody
ANCA: Anti-neutrophil cytoplasmic antibody
ANKH: ANKH inorganic pyrophosphate transport regulator
ANTRX2: Anthrax toxin receptor 2
AP: Alkaline phosphatase
BMP: Bone morphogenetic protein
BUN: Blood urea nitrogen
CAPS: Cryoporin-associated periodic syndrome
CCL18: CC-chemokine ligand 18
CECR1: Cat eye syndrome chromosome region
CIAS1: Cold-induced autoinflammatory syndrome 1
CINCA: Chronic infantile neurological, cutaneous, and articular syndrome
CMG2: Capillary morphogenesis gene 2
COL1A1: Collagen type 1 alpha 1
CPTII: Carnitine palmitoyltransferase 2
CPPD: Calcium pyrophosphate dehydrate
DM: Dermatomyositis
DMARD: Disease-modifying anti-rheumatic drug
DNASE1L3: Deoxyribonuclease 1 like 3
DVT: Deep vein thrombosis
FBN1: Fibrillin 1
FCGR2A/3A: Fc fragment of IgG receptor 2A/3A
GAG: Glycosaminoglycan
GALNS: Galactosamine (N-acetyl)-6-sulfatase
GBA1: Glucocerebrosidase beta
GCSF: Granulocyte colony-stimulating factor
GI: Gastrointestinal
GNE: Glucosamine (UDP-N-acetyl)-2-epimerase/N-acetylmannosamine kinase
HDAC9: Histone deacetylase 9
HLA: Human leukocyte antigene
HMGCoA: 3-Hydroxy-3-methylglutaryl-coenzyme A
HPGD: Hydroxyprostaglandin dehydrogenase
IDS: Iduronate 2-sulfatase
IL: Interleukin
ILAR: International League of Associations for Rheumatology
ILD: Interstitial lung disease
ING5: Inhibitor of growth family member 5
IOMID: Infantile-onset multisystem inflammatory disease
JIA: Juvenile idiopathic arthritis
KIT: KIT proto-oncogene receptor tyrosine kinase
MIF: Macrophage migration inhibitory factor
HLA: Human leukocyte antigene
MAS: Macrophage activation syndrome
MDS: Myelodysplastic syndrome
MEFV: Mediterranean fever
MIF: Macrophage migration inhibitory factor
MMP3: Matrix metalloproteinase-3
MPO: Myeloperoxidase
MVK: Mevalonate kinase
NF-κB: Nuclear factor kappa-light-chain-enhancer of activated B-cells
NOD2: Nucleotide binding oligomerization domain containing 2
NOMID: Neonatal onset multisystem inflammatory disease
NSAID: Nonsteroidal anti-inflammatory drug
PAH: Pulmonary arterial hypertension
PFKM: Muscle phosphofructokinase
PGYM: Muscle glycogen phosphorylase
PIK3CA: Phosphatidylinositol-4,5-bisphosphate 3-kinase catalytic subunit Α
PM: Polymyositis
PPD/PPRD: Progressive pseudorheumatoid dysplasia
PSMB8: Proteasome subunit beta 8
PSTPIP1: Proline-serine-threonine phosphatase interacting protein 1
PTX3: Pentraxine 3
RF: Rheumatoid factor
RA: Rheumatoid arthritis
SAA: Serum amyloid A
SAPHO: Synovitis-acne-pustulosis-hyperostosis-osteitis-syndrome
SEDT-PA: Spondyloepiphyseal dysplasia tarda with progressive arthropathy
SLE: Systemic lupus erythematosus
TGFB1: Transforming growth factor
TIMP1: Tissue inhibitor of metalloproteinase
TNFi: Tumor necrosis factor inhibitor
TNFRSF: Tumor necrosis factor receptor superfamily
VEGF: Vascular endothelial growth factor
WBC: White blood cell
WISP3: WNT1 inducible signaling pathway protein
